# Accuracy and cost comparison of 3D-printed guides in complex spinal deformity correction: direct vs indirect design

**DOI:** 10.3389/fbioe.2025.1611998

**Published:** 2025-05-30

**Authors:** Wei Yang, Wei Guo, Wen-Jun Wu, Rong Ma, Zemin Wang, Honglai Zhang, Wanzhong Yang, Zhaohui Ge

**Affiliations:** ^1^ Department of Orthopedic, General Hospital of Ningxia Medical University, Yinchuan, China; ^2^ First Clinical Medical College, Ningxia Medical University, Yinchuan, China; ^3^ People’s Hospital of Ningxia Hui Autonomous Region, Yinchuan, Ningxia, China

**Keywords:** 3D printing, spinal deformity, screw guide, osteotomy guide, patient-specific design, design variations, spine surgery

## Abstract

**Background:**

The treatment of complex spinal deformities poses significant challenges, as the placement of pedicle screws and the execution of osteotomies within deformed vertebral structures carry an elevated risk of neurological complications. Numerous techniques have been developed to enhance the safety and accuracy of pedicle screw placement and osteotomies. Among these techniques, patient-specific guides, which feature pre-defined and pre-validated trajectories, present an attractive solution for achieving precision in screw placement and osteotomies.

**Methods:**

CT scan data (DICOM format) from 10 patients with complex and severe spinal deformities were selected. Full spinal reconstruction was performed using Mimics, CAD, and E−3D software. Two different types of screw placement and osteotomy guides were designed: direct (using a larger aperture design to allow direct screw placement) and indirect (using a K-wire or 2.5 mm drill bit to preset the screw path before screw placement). Screw placement and osteotomy were simulated using 3D-printed spinal models and guides. Post-operative CT scans were performed on the models and compared with pre-operative designs to evaluate the accuracy, efficiency, cost, and clinical practicality of different guides during screw placement and osteotomy.

**Results:**

This study included 10 patients with complex spinal deformities (Five males and five females, with an average age of 37 years), covering five diagnostic types such as neurofibromatosis and adult idiopathic spinal deformity. Nine cases of Vertebral Column Resection (VCR) and one case of pedicle subtraction osteotomy (PSO) were performed. Experimental data showed no statistically significant differences between the direct and indirect guide groups in terms of pedicle screw placement accuracy (95.97% vs. 94.63%), coronal osteotomy accuracy (ROED 96.69% vs. 98.68%), and sagittal osteotomy accuracy (94.24% vs. 96.86%) (P > 0.05). However, the digital preparation efficiency of the direct guide group was significantly lower than that of the indirect group, with a 33.2% increase in single guide design time and a 44.6% increase in printing time (P < 0.001), resulting in a 35.8% increase in total design time (P = 0.026). There were no significant differences between the two groups in screw placement time (4.24 vs. 4.79 min), osteotomy time (37.15 vs. 36.56 min), and material cost ($268.25 each). The results indicate that both guide techniques can achieve precise orthopedics, but the indirect guide has advantages in clinical transformation efficiency.

**Conclusion:**

Both direct and indirect 3D-printed guides can optimize screw implantation and complex osteotomy procedures, improving the accuracy of pedicle screw placement and osteotomy. However, the direct guide group has clinical limitations such as extended design cycles, increased printing time, and expanded surgical field exposure.

## 1 Introduction

Severe complex spinal deformities often present with intricate anatomical features, spinal rigidity, and significant kyphosis or scoliosis, which may result in neurological impairment, limb disability, and even life-threatening complications (Ding et al., 2023). Advances in surgical instruments, neuromonitoring technology, and anesthesia have brought transformative progress in treating these severe deformities, offering new hope to patients with limited options and markedly improving their quality of life (Kim et al., 2022; 2016). However, the correction of severe, rigid scoliosis (Cobb angle >80° and flexibility <25%) (Liu et al., 2017) poses substantial challenges for pedicle screw placement, vertebral osteotomies, alignment correction, and decompression, given the complex spinal morphology. These procedures are associated with risks such as spinal cord ischemia, nerve injury, and arterial rupture. Inaccurate screw placement rates are reported to be as high as 29.1% (Sarlak et al., 2009)—Additionally, a study by Suk et al. (Kim et al., 2012) reported a postoperative complication rate of 40.3% in 233 patients undergoing posterior vertebral column resection (PVCR) for severe spinal deformities, with complication risk closely associated with deformity severity, osteotomy levels, osteotomy type, and kyphosis correction rate (Chen et al., 2020). Since the degree of preoperative deformity is often challenging to manage, comprehensive perioperative assessment and tailored surgical planning—focusing on precise implant placement and optimal osteotomy design—are crucial to achieving successful outcomes.

Since its development in the 1980s (Garg and Mehta, 2018; Pugliese et al., 2018), 3D printing (3DP) technology has shown broad applications in orthopedic surgery. Beyond its role as an educational tool for training and doctor-patient communication, 3DP has been widely adopted in preoperative planning, anatomical visualization, and customized implant design (Lopez et al., 2021; Sheha et al., 2019; Yang et al., 2015; Zamborsky et al., 2019). For patients with spinal deformities, 3DP technology has been found to assist in pedicle screw placement and osteotomies, facilitating surgical planning, enhancing screw placement accuracy, reducing operative time, minimizing blood loss, and preventing complications (Garg et al., 2019; Izatt et al., 2007; Pan et al., 2023). The existing literature reports on two typical forms of 3D-printed navigational templates for spinal orthopedic surgery: the direct navigation template, which utilizes a larger aperture design to facilitate direct screw placement (direct method), and the indirect navigation template, which employs a smaller aperture in conjunction with a Kirschner wire or a 2.5 mm drill bit to prepare the screw path before screw placement (indirect method). Surgeons typically choose one of these methods based on their proficiency with design software and personal preference for clinical application and validation. However, an evaluation system has not been established for key indicators such as screw placement accuracy, osteotomy precision, design cost, and economic suitability, limiting the scientific selection for clinical applications.

Therefore, this study aims to systematically evaluate two different screw placement and osteotomy guide plate systems based on 3D-printed spinal models and postoperative CT scan data. The evaluation will cover multiple dimensions, including screw placement accuracy, osteotomy precision, clinical suitability, and economic indicators (such as material cost and labor time consumption). The goal is to provide objective data support for clinical selection of navigational templates under different medical resource conditions.

## 2 Materials and methods

This study was approved by our institutional review board, and informed consent was obtained for the use of patient data. We included 10 patients diagnosed with complex severe spinal deformities, including neuromuscular scoliosis (NF-1), tuberculosis kyphosis (TB), adult idiopathic scoliosis (ADIS), congenital scoliosis (CS), and ankylosing spondylitis (AS). Severe deformity was defined as kyphotic/scoliotic Cobb angles exceeding 80° with spinal flexibility less than 25%.

### 2.1 Digital reconstruction of the spine

**FIGURE 1:**
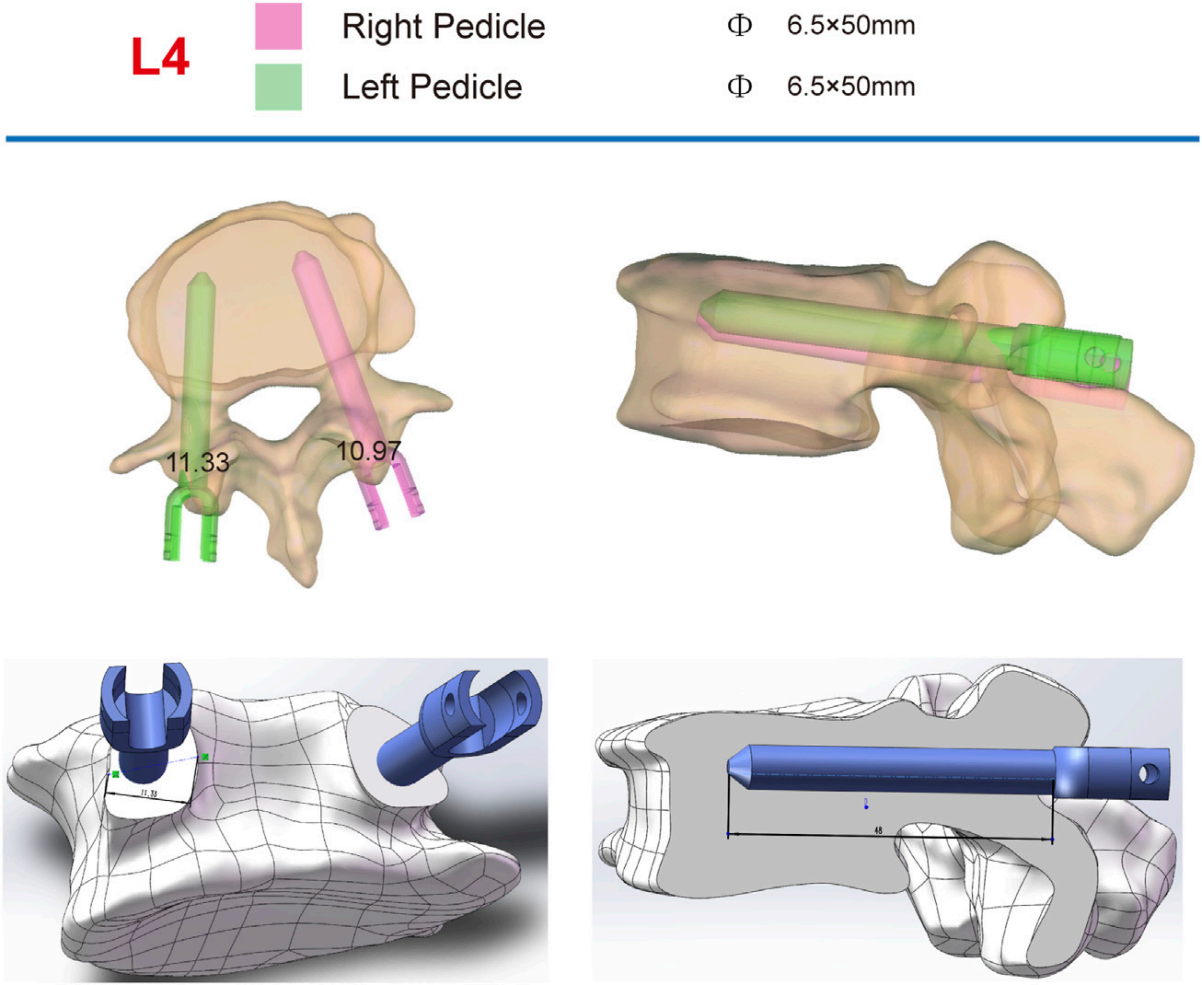
Individual analysis of each vertebra to determine rotation and pedicle size, with the selection of the most appropriate screw size based on measurement outcomes.

CT scans of each patient (SOMATOM Sensation 16, Siemens AG, Forchheim, Germany, matrix: 512 × 512, slice thickness: 0.625 mm) were obtained and converted to DICOM format. These data were imported into Mimics Innovation Suite 21.0 (Materialise, Leuven, Belgium) for three-dimensional (3D) reconstruction. The generated 3D model was then transferred to SolidWorks 2022 CAD (Dassault Systèmes Americas Corp, Waltham, MA, United States) for individualized analysis of each vertebral segment, determining vertebral rotation, translation, and pedicle dimensions. The most suitable screw size and length were selected based on each segment’s specific requirements, excluding segments deemed unsuitable for fixation (Figure 1).

### 2.2 Construction of pedicle screw navigation templates

**FIGURE 2:**
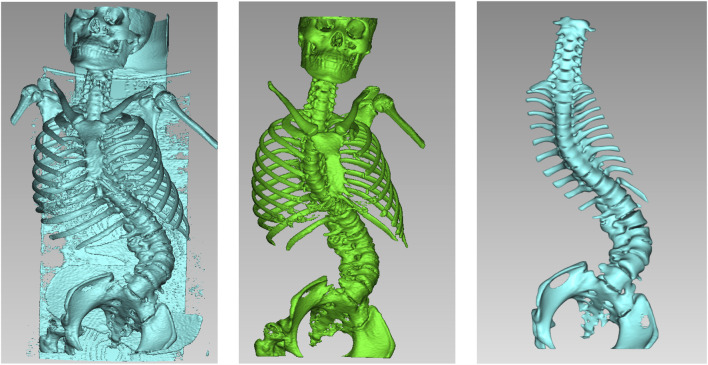
The original model was generated by applying threshold segmentation, mass separation, seed point labeling, and 2D editing to the patient’s DICOM images in sequence.

**FIGURE 3:**
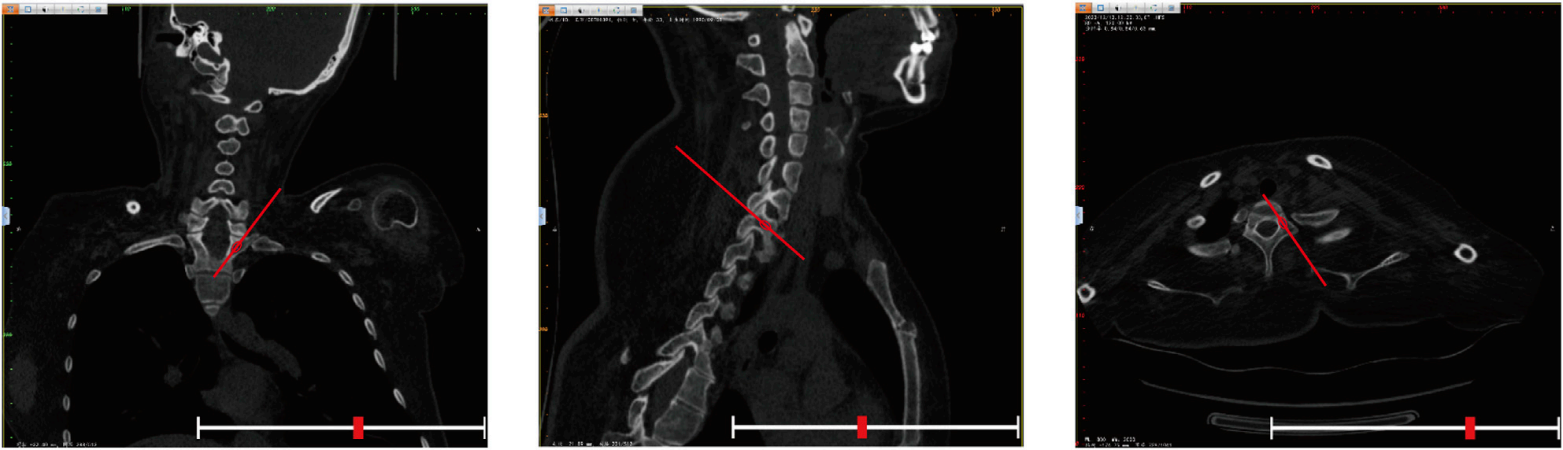
Adjustment of screw trajectory angles and paths in the three-dimensional viewing windows (coronal, sagittal, and axial). The red line indicated the angle of the screw insertion path, while the red ellipse displayed the real-time position of the screw.

**FIGURE 4:**
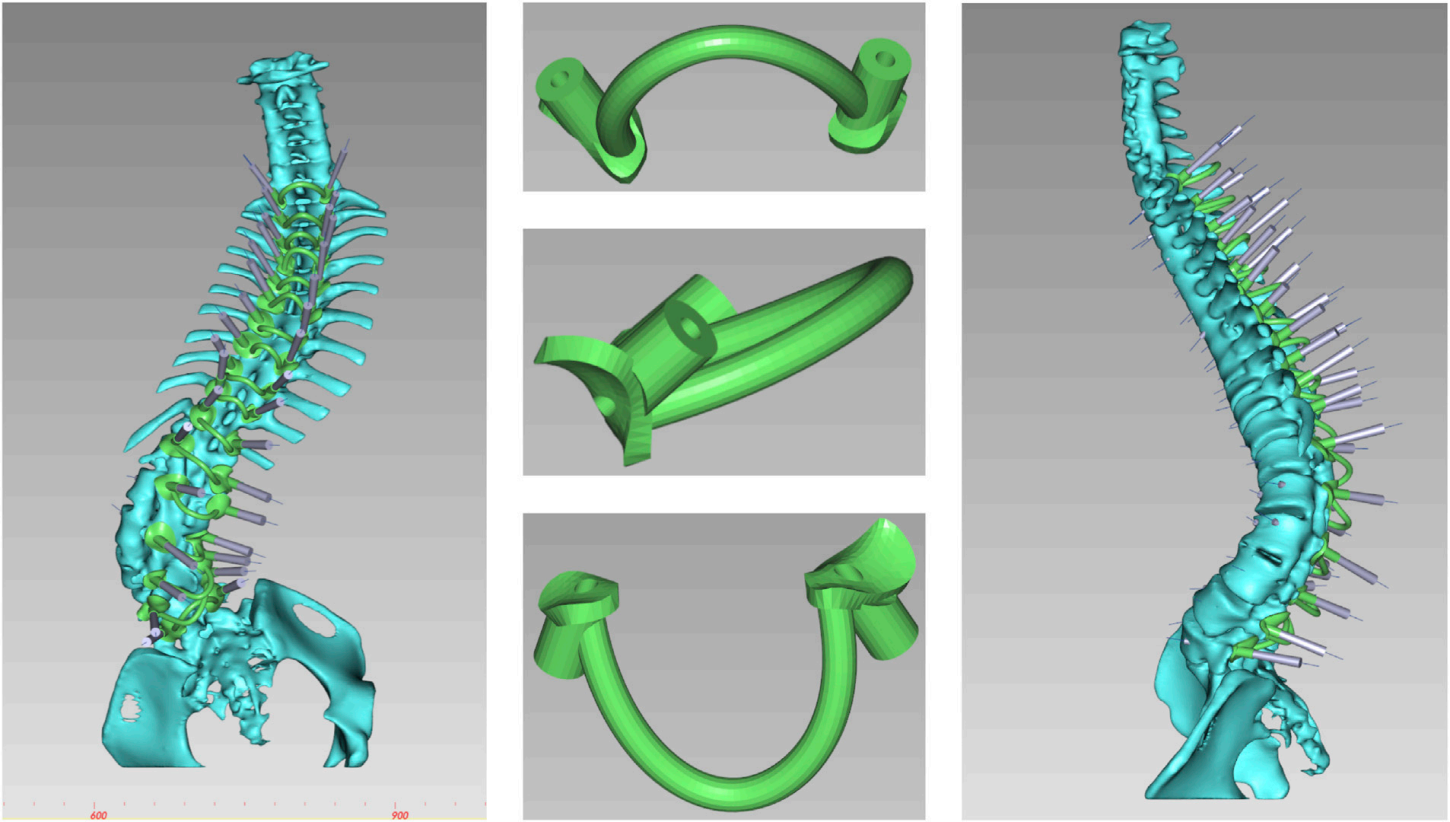
Indirect placement of pedicle screw guidance templates. The parameters of this guide were: height 15 mm, inner diameter 3 mm, wall thickness 2.5 mm, allowing a drill bit with an outer diameter of 2.5 mm to pass through smoothly.

**FIGURE 5:**
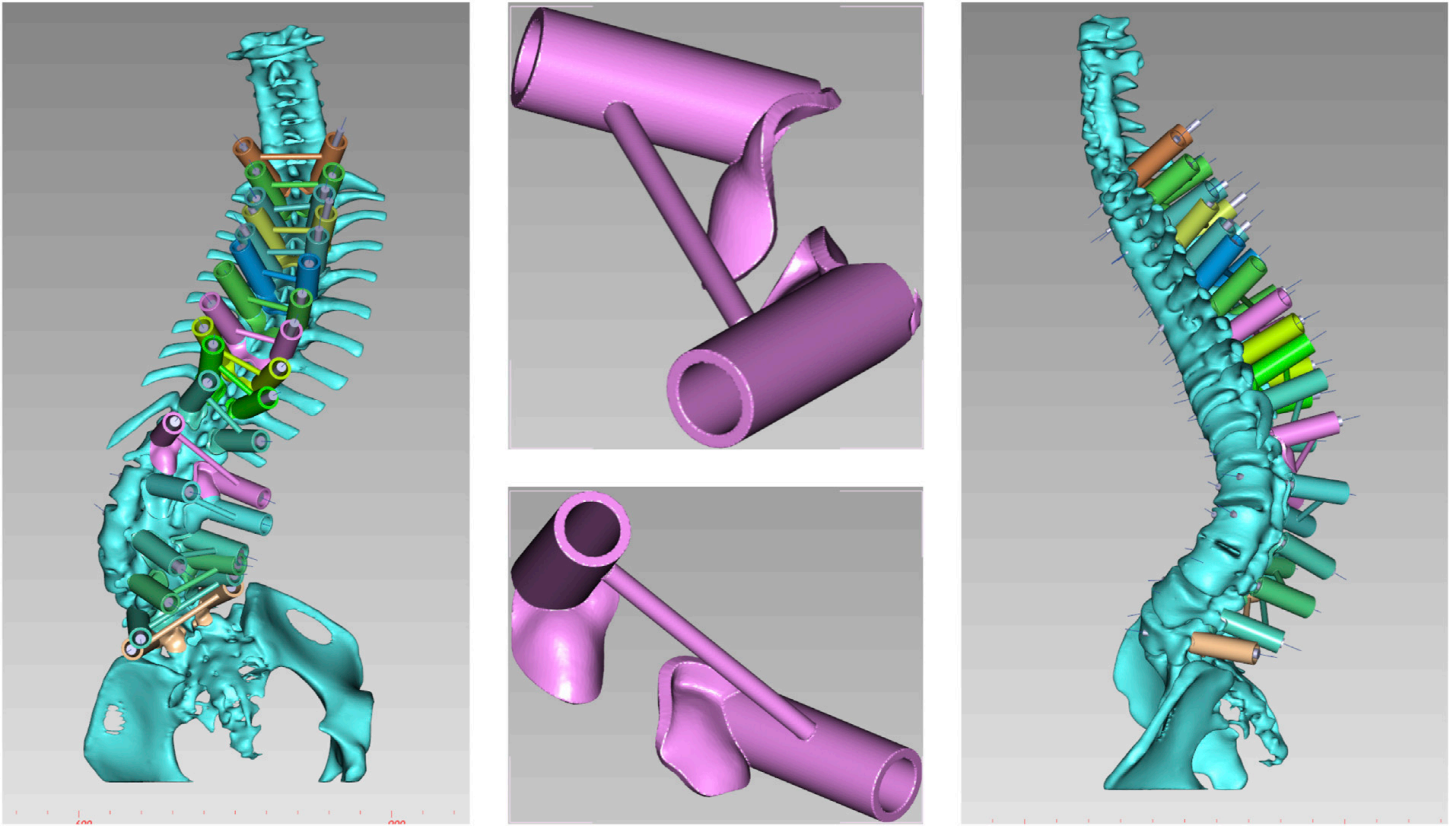
Direct placement of pedicle screw guidance templates. The guide parameters were as follows: height 50 mm, inner diameter 13 mm, wall thickness 2.5 mm, designed to ensure smooth placement of pedicle screws (UPASS/Premier 5.5 mm rod system).

(1) Original Model: The CT data were imported into E−3D Medical Modeling-Design-Simulation software (Central South University Digital Medicine and Virtual Reality Research Center, V21.10). An original model was developed through threshold segmentation, region-growing techniques, noise removal, contour refinement, and 2D editing, isolating specific anatomical landmarks (Figure 2). (2) Screw Path Planning: Screw paths were defined on the model by marking trajectories in the coronal, sagittal, and axial planes, ensuring paths did not breach pedicle cortices. For pedicles narrower than 3.0 mm, paths avoided the medial cortex to prevent canal intrusion. Vertebrae lacking pedicles were excluded, and screw dimensions were adjusted according to measurements (Figure 3). (3) Baseplate Generation: Using “Path Extraction,” vertebral surface landmarks were marked to create template anchoring points, forming a baseplate model. (4) Template Bridging: Bridge points were created *via* “Triple or Free Bridging,” adjusting bridge placement and shape to minimize surgical interference. (5) Indirect Screw Guide Template: Guide templates were generated with a 15 mm height, 3 mm inner diameter, and 2.5 mm wall thickness to allow a 2.5 mm drill passage (Figure 4). (6) Direct Screw Guide Template: Similar steps were followed but with dimensions suited to accommodate thicker screws (UPASS/Premier 5.5 mm screw-rod system), using a height of 50 mm, inner diameter of 13 mm, and wall thickness of 2.5 mm. To ensure structural integrity, part of the base was reinforced, with minimum diameters designed to match screw tail widths. Bridging was completed *via* CAD post-template generation to minimize intraoperative interference. (Figure 5).

### 2.3 Construction of osteotomy guide templates

**FIGURE 6:**
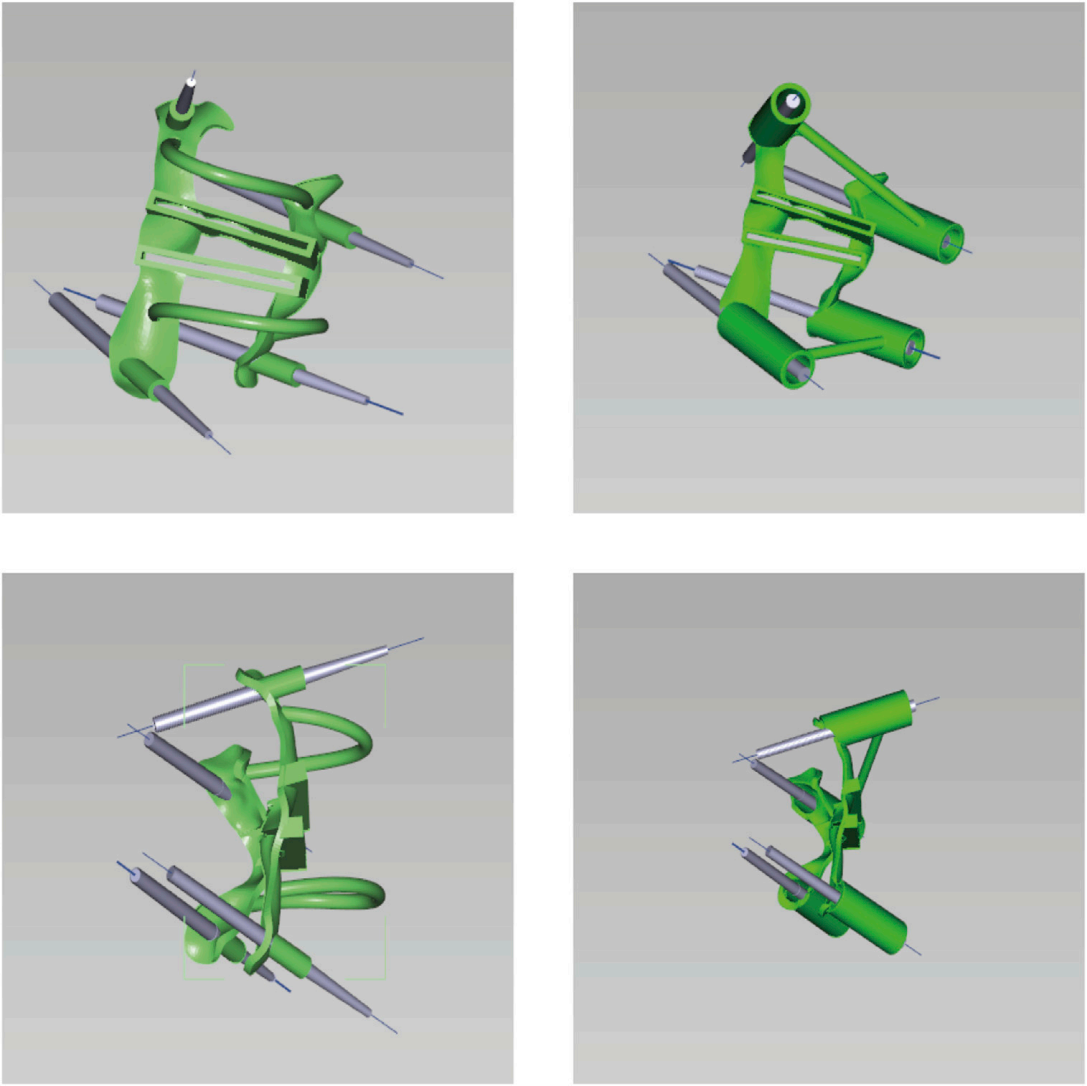
Anteroposterior and lateral views of the osteotomy guide. The width of the cutting notch was set to 1 mm, adjusted according to the specifications of the ultrasonic osteotome.

The osteotomy guide consisted of three sections: the pedicle screw guide for adjacent segments, the lamina-fitted surface, and the osteotomy trajectory guide. Based on prior adjustments, trajectories for screws in adjacent segments were finalized. The guide base for lamina fit was generated using “Path Extraction” to select anatomical landmarks within the exposure area. After defining the osteotomy angle, a cutting plane was set, and adjusted with a brush to simulate the cut, and a 1 mm-wide slot for an ultrasonic bone saw was created to ensure sufficient depth without spinal cord risk. The osteotomy slot was integrated with the base (Figure 6).

### 2.4 3D printing

STL files of the spine model, screw navigation templates, and osteotomy guides were exported to preprocessing software (Polydevs, BPC) with a slice thickness of 0.1 mm for model accuracy. Models and templates were printed on an SLA photopolymerization printer (UnionTech Lite 600, Shanghai Union Technology Corporation) with Syn 80 photopolymer resin, achieving a printing accuracy deviation of less than 0.2% per 10 cm.

### 2.5 Simulated surgery

The indirect screw placement/osteotomy group positions the guiding template on the lamina and spinous process according to designated markings, ensuring alignment with the patient’s spinal model. A 2.5 mm drill bit is inserted through the template into the pedicle, and drilling begins at a controlled pace. Upon reaching the predetermined depth, the drill is retracted, and a screw is inserted. The osteotomy template is secured, and a 1 mm high-speed bur is used to incise along the template’s groove to excise the lamina. After removing the template, screws are placed in adjacent segments, followed by either pedicle subtraction osteotomy (PSO) or vertebral column resection (VCR) as indicated by the surgical incision.

**FIGURE 7:**
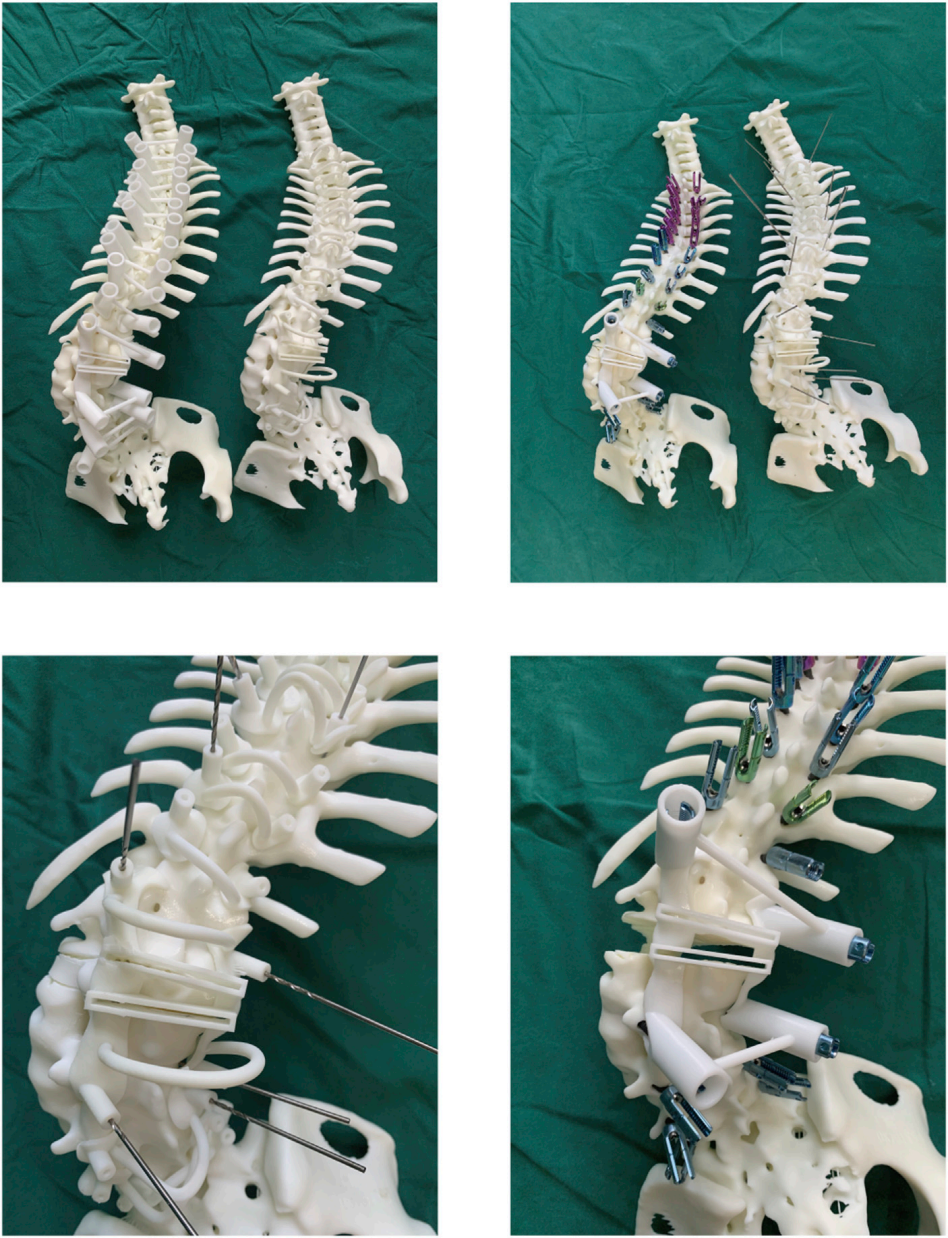
Navigation templates perfectly match the vertebrae and simulate screw placement and osteotomy on the 3D-printed model.

The direct pedicle screw guiding template is positioned on the lamina and spinous process surfaces as marked. An assistant applies pressure to ensure stable contact. Screws are inserted directly, alternating sides, and the guiding plate is then removed. The osteotomy guiding template is secured, and pedicle screws are placed in adjacent segments. A 1 mm high-speed bur is used to excise the lamina along the groove of the 3D-printed template. Afterward, the guiding template is removed, and either PSO or VCR osteotomy is performed according to the surgical incision (Figure 7).

### 2.6 Data collection and statistical analysis

**FIGURE 8:**
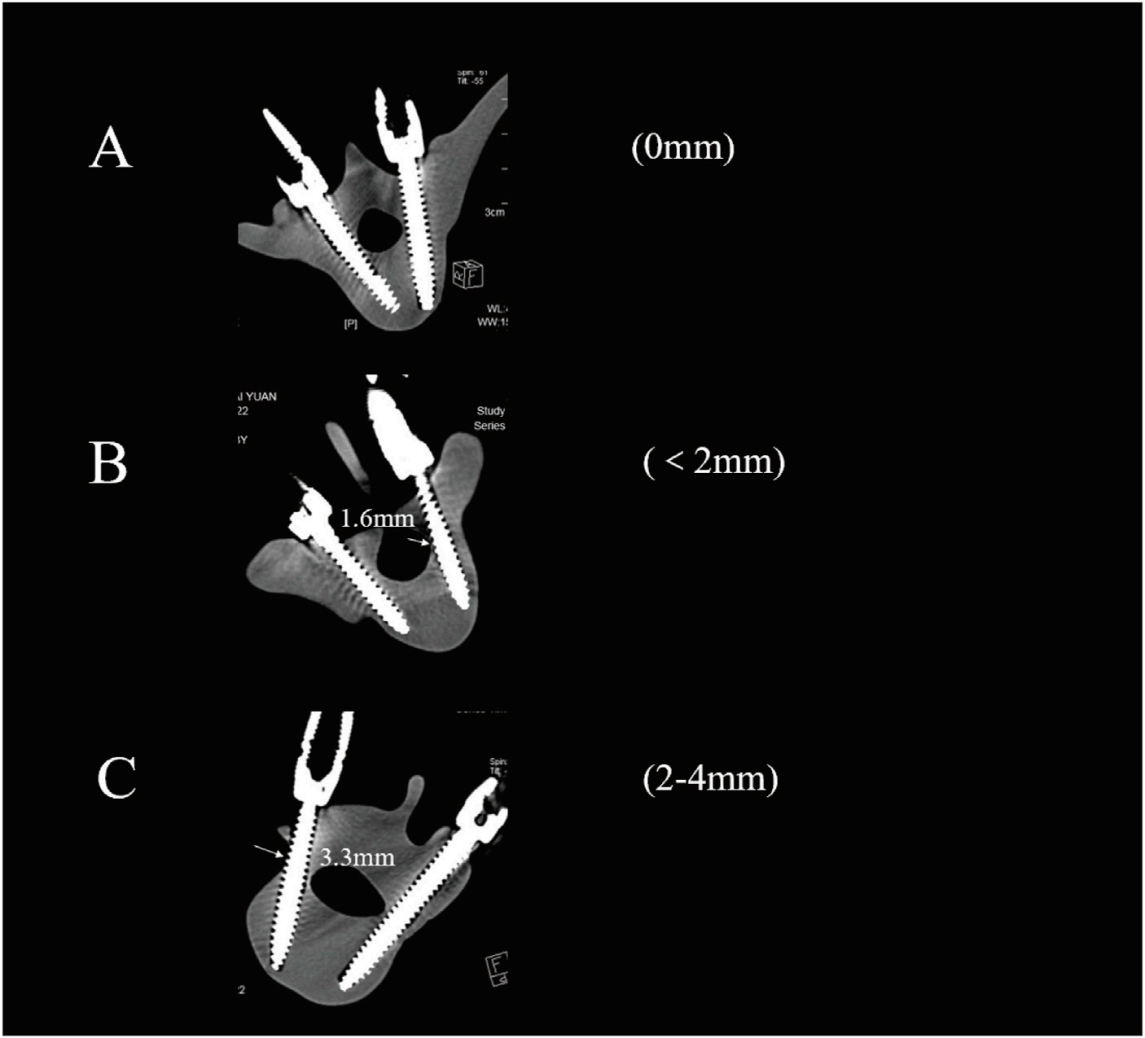
Axial CT scans classify pedicle screws (based on Gertzbein - Robbins). Grade **(A)** screws are fully within the pedicle (0 mm offset); Grade **(B)** have < 2 mm cortical penetration; Grade **(C)** have 2–4 mm penetration.

**FIGURE 9:**
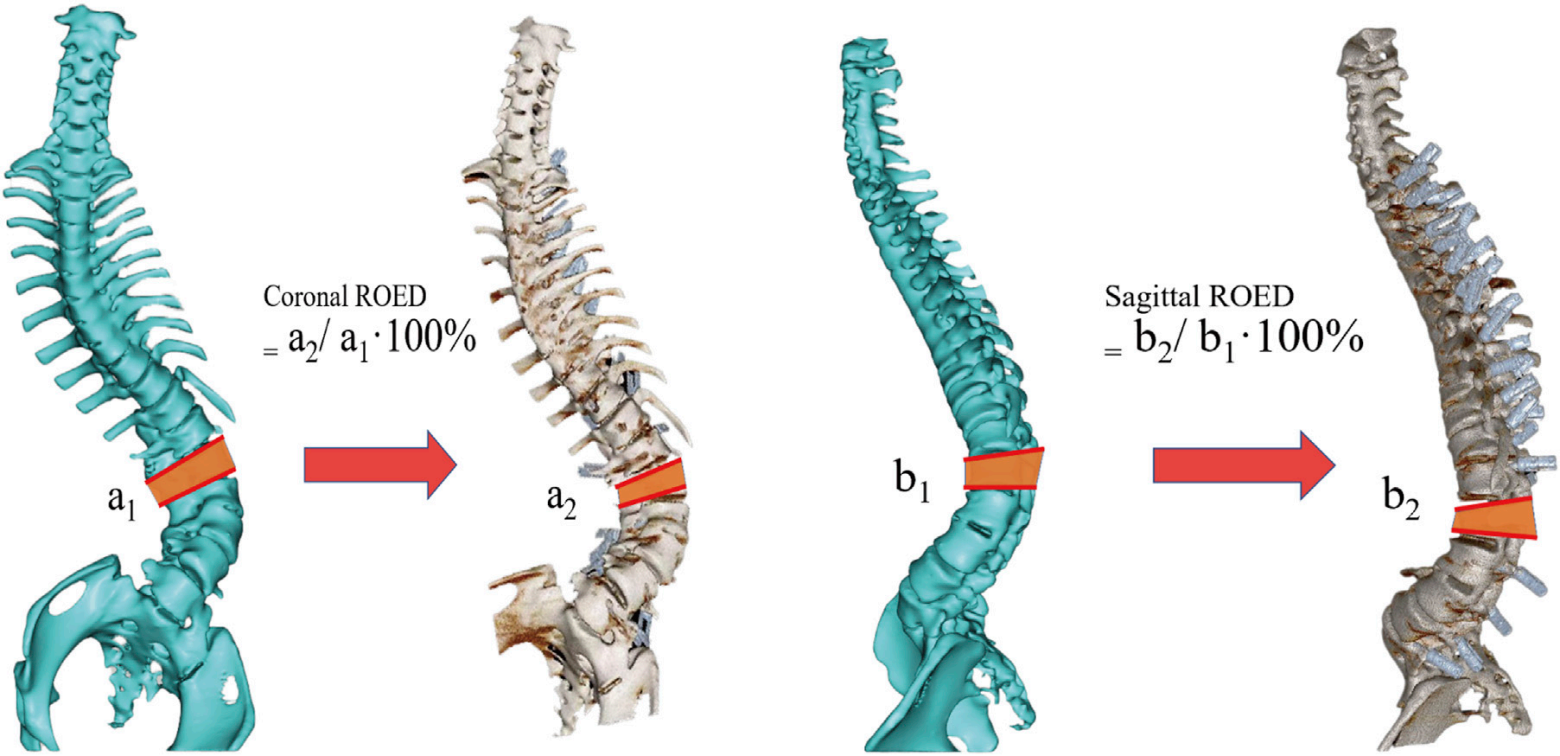
Schematic diagram of the osteotomy execution-to-design ratio calculation. It shows coronal ROED (a_2_/a_1_⋅100%) and sagittal ROED (b_2_/b_1_⋅100%), where **a_1_**, **a_2_**, **b_1_** and **b_2_** are the designed and executed parameters in coronal and sagittal planes, respectively.

The Gertzbein-Robbins classification (Gertzbein and Robbins, 1990) assesses screw placement precision, defined by the absence of vertebral pedicle wall destruction and acceptability indicated by cortical perforation of less than 2 mm (Figure 8). Osteotomy accuracy is measured by the Rates of Osteotomy Execution and Design (Ding et al., 2023) (ROED, %), comparing the executed to intended coronal/sagittal Cobb angles (refer to Figure 9).

Data analysis was conducted using IBM SPSS version 28.0 (IBM, New York, Unites States). The Shapiro-Wilk test assessed the normality of continuous variables, which are reported as mean ± standard deviation for normal distributions and as median and interquartile range for non-normal distributions. Paired t-tests were used for measurement data, while count data were analyzed with the Mann-Whitney U test or chi-square test, considering a two-sided P value of less than 0.05 statistically significant.

## 3 Result

### 3.1 Patient demographics and surgical parameters

The study included models from 10 patients with spinal deformities, with a mean age of 37 years (range: 10–68 years), consisting of five females and five males. Diagnoses included neurofibromatosis type 1 (NF-1, 2 cases), ankylosing spondylitis (AS, 1 case), spinal tuberculosis (TB, 2 cases), congenital scoliosis (CS, 3 cases), and adult idiopathic scoliosis (ADIS, 2 cases). Deformities were categorized as kyphoscoliosis (7 cases), isolated kyphosis (2 cases), and isolated scoliosis (1 case). Surgical interventions included vertebral column resection (VCR, 9 cases) and pedicle subtraction osteotomy (PSO, 1 case), with four patients undergoing multilevel VCR procedures. The fusion levels spanned from T1 to S1, with osteotomies concentrated in the thoracolumbar region (Table 1).

**TABLE 1:** General and operative parameters of included patients.

No.	Gender	Age	Diagnosis	Deformity	Instrumented	Osteotomy	Osteotomy
				Type	Segments	Type	Position
1	F	33	NF-1	Kyphoscoliosis	T2-L5	VCR	L1
2	M	61	AS	Kyphosis	T9-L4	PSO	L2
3	F	56	TB	Kyphosis	T10-S1	VCRs	L1-L4
4	F	11	CS	Kyphoscoliosis	T7-L4	VCR	T12
5	M	68	TB	Kyphosis	T2-L3	VCRs	T5-T12
6	M	35	ADIS	Kyphoscoliosis	T3-L4	VCR	T10
7	M	27	ADIS	Kyphoscoliosis	T2-L4	VCRs	T12/L1
8	F	10	CS	Scoliosis	T3-L4	VCR	T11
9	F	14	CS	Kyphoscoliosis	T1-L1	VCRs	T8/T9
10	M	55	NF-1	Kyphoscoliosis	T5-L5	VCR	L2

NF-1, Neurofibromatosis type 1 scoliosis; AS, ankylosing spondylitis; TB, tuberculosis; CS, congenital scoliosis; ADIS, adult idiopathic scoliosis; VCR, vertebral column resection; PSO, Pedicle Subtraction Osteotomy.

### 3.2 Accuracy of pedicle screw placement

149 pedicle screws were placed in each of the two groups of models: 125 screws in the direct group and 121 screws in the indirect group achieved accurate positioning, with acceptable placement rates of 95.97% *versus* 94.63%, respectively. Pedicle cortical breaches occurred in both groups, though most were within 2 mm (Type B, 18/24 in the direct group vs 20/28 in the indirect group). Screw misplacement rates were 4.03% for the direct group and 5.37% for the indirect group. No screws in either group were classified as Type D or E (Figure 2). No statistically significant differences in screw accuracy or breach distribution were found between the two groups (P > 0.05) (Table 2).

**TABLE 2:** Accuracy of Screws insertion.

Gertzbein-robbins screw classification	Direct group (n = 149)	Indirect group (n = 149)	*P* Value
A	125	121	0.528
B	18	20	
C	6	8	
D/E	0	0	
Correctly Placed (A and B)	143 (95.97%)	141 (94.63%)	0.784
Incorrectly Placed (C--E)	6 (4.03%)	8 (5.37%)	

Direct Group, direct 3D printed guide plate placement of pedicle screw set; Indirect Group, indirect 3D printed guide plate placement of pedicle screw set.

### 3.3 Accuracy of osteotomy angle design and execution

#### 3.3.1 Coronal Plane

The planned osteotomy angles ranged from 30° to 54°. The actual angles achieved in the direct group ranged from 27° to 56°, with ROED values between 90% and 103.7% (mean: 96.69% ± 4.37%). In the indirect group, the actual angles achieved ranged from 30° to 55°, with ROED values between 92.11% and 105% (mean: 98.68% ± 4.78%).

#### 3.3.2 Sagittal plane

**FIGURE 10:**
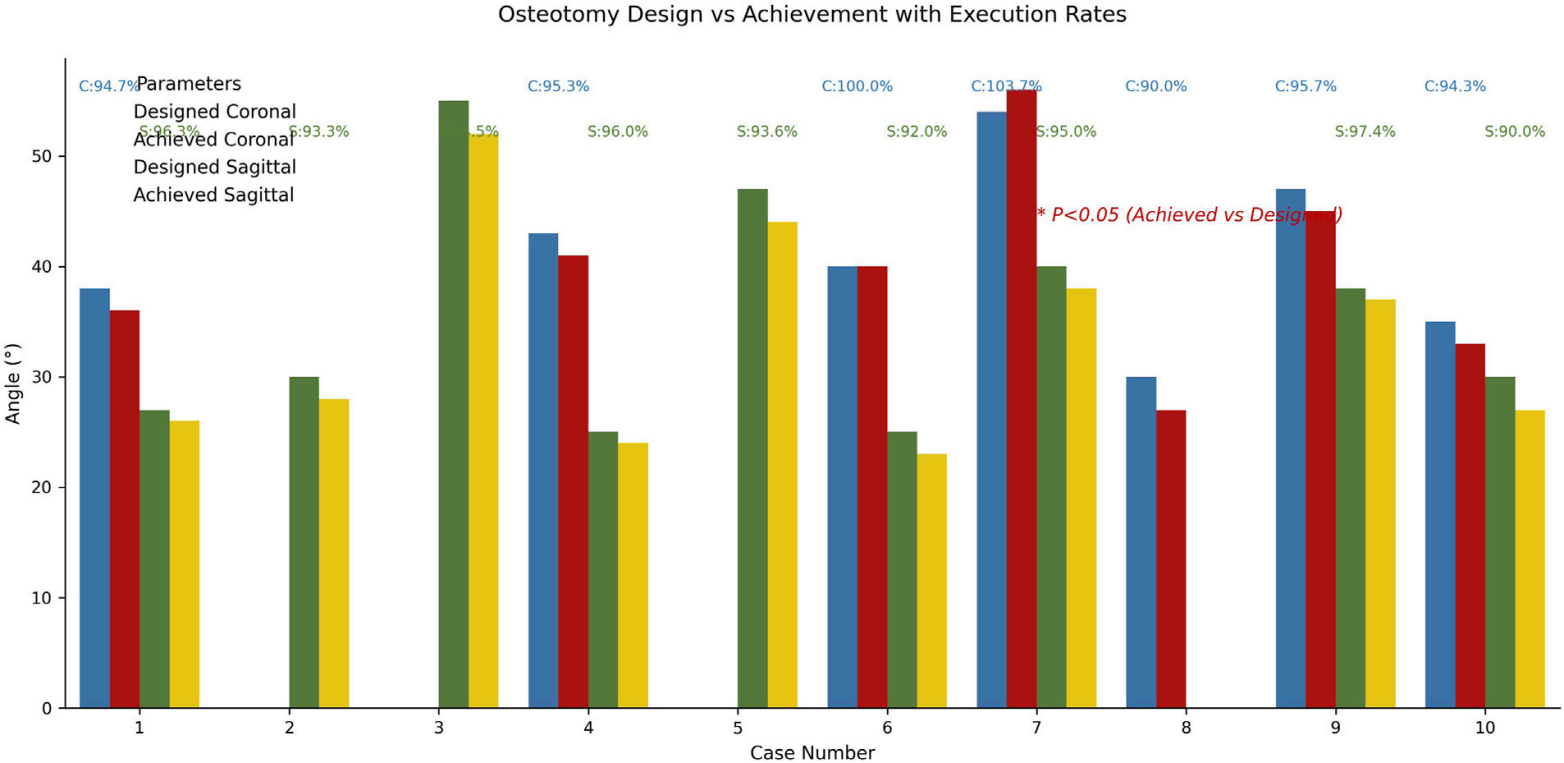
Comparative analysis diagram of osteotomy surgery design and implementation effects. Blue/red histograms represent coronal plane design/actual angles, green/gold represent sagittal plane angles. In both planes, there was no significant difference in osteotomy accuracy between the direct and indirect groups (*P* = 0.433, *P* = 0.156).

The planned angles ranged from 25° to 55°. The actual angles achieved in the direct group ranged from 24° to 52°, with a mean ROED of 94.24% ± 2.29% (range: 90%–97.37%). In the indirect group, the actual angles achieved ranged from 23° to 54°, with a mean ROED of 96.86% ± 4.64% (range: 90%–103.7%). There were no significant differences in osteotomy precision between the direct and indirect groups in both the coronal and sagittal planes (P = 0.433, P = 0.156), demonstrating consistent execution accuracy for both methods (Tables 3, 4; Figure 10).

**TABLE 3:** Osteotomy design parameters for enrolled patients.

No.	Scoliosis	Kyphosis	Designed osteotomy Angle (°)		Achieved osteotomy Angle (°) (A(B))		ROED (%) (A(B))	
			Coronary	Sagittal	Coronary	Sagittal	Coronary	Sagittal
1	97	82	38	27	36 (35)	26 (28)	94.74 (92.11)	96.3 (103.7)
2	—	88	—	30	—	28 (30)	—	93.33 (100)
3	—	119	—	55	—	52 (54)	—	94.54 (98.18)
4	99	94	43	25	41 (41)	24 (23)	95.35 (95.35)	96 (92)
5	—	101	—	47	—	44 (45)	—	93.62 (95.74)
6	108	97	40	25	40 (42)	23 (25)	100 (105)	92 (100)
7	119	117	54	40	56 (55)	38 (36)	103.7 (101.85)	95 (90)
8	88	—	30	—	27 (30)	—	90 (100)	—
9	103	114	47	38	45 (48)	37 (35)	95.74 (102.13)	97.37 (92.11)
10	94	105	35	30	33 (33)	27 (30)	94.29 (94.29)	90 (100)

A, direct group; B, indirect group; ROED, Ratio of osteotomy execution to design.

**TABLE 4:** Comparison of osteotomy accuracy.

Clusters	ROED (%) ( $\bar{x}$ ± s)	
	Coronary	Sagittal
Direct Group	96.69 ± 4.37	94.24 ± 2.29
Indirect Group	98.68 ± 4.78	96.86 ± 4.64
*P* value	0.433	0.156

### 3.4 Design, operative time, and cost

**FIGURE 11:**
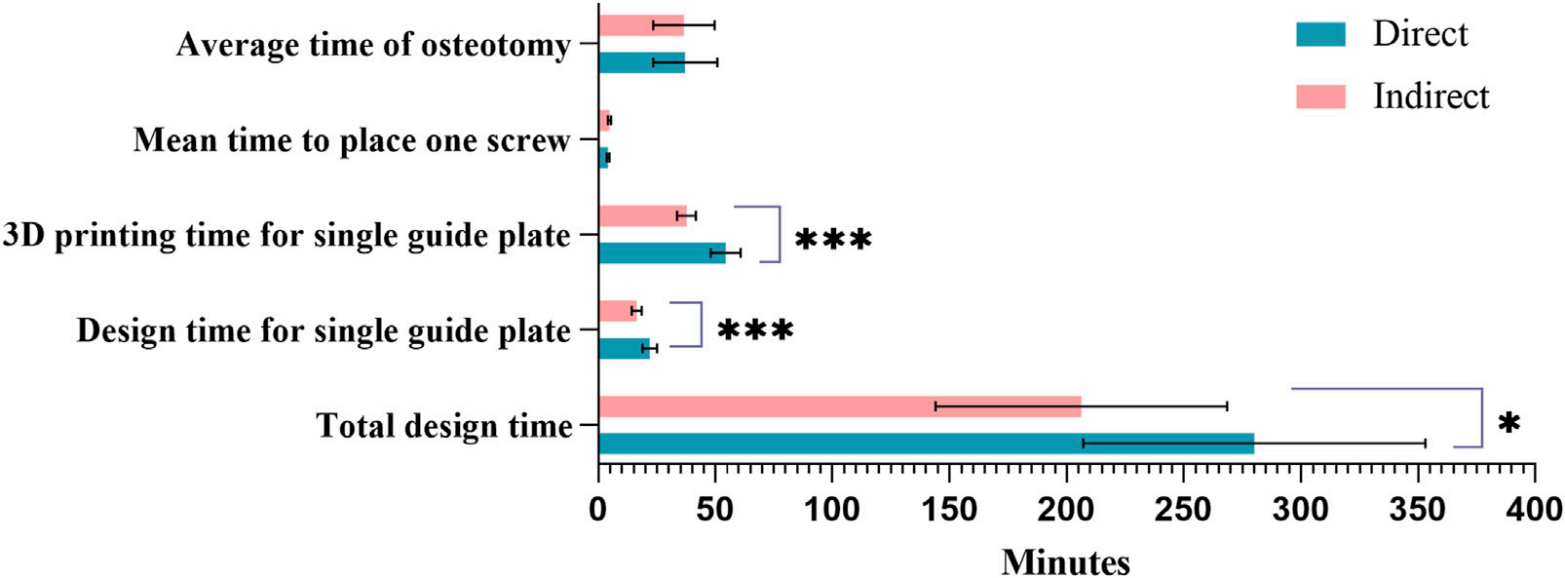
Comparison of design costs between the two groups. Sapphire blue represents the direct group, pink the indirect group; *Indicates *P* < 0.05; ***Indicates *P* < 0.001.

The direct group showed significantly higher times in design, template preparation, and overall surgical time compared to the indirect group. Total Design Time: The direct group averaged 280.0 ± 73.03 min *versus* 206.2 ± 62.25 min in the indirect group (P = 0.026). Template Design Time per Unit: The direct group averaged 21.99 ± 3.16 min, compared to 16.52 ± 2.12 min in the indirect group (P < 0.001). 3D Printing Time per Template: The direct group averaged 54.50 ± 6.39 min, while the indirect group averaged 37.69 ± 3.99 min (P < 0.001). Although the direct group required more time for design and printing, no significant differences were found in other surgical parameters, such as mean screw placement time (4.24 ± 0.54 vs. 4.79 ± 0.66) and mean osteotomy time (37.15 ± 13.64 vs. 36.56 ± 13.10). The average printing cost was consistent between groups, at $268.25 (Table 5; Figure 11).

**TABLE 5:** Comparison between the design costs of the two groups.

Parameters	Direct group (n = 10)	Indirect group (n = 10)	*P* Value
Total design time (minutes)	280.0 ± 73.03	206.2 ± 62.25	0.026
Design time for single guide plate (minutes)	21.99 ± 3.16	16.52 ± 2.12	<0.001
3D printing time for single guide plate (minutes)	54.50 ± 6.39	37.69 ± 3.99	<0.001
Mean time to place one screw (minutes)	4.24 ± 0.54	4.79 ± 0.66	0.056
Average time of osteotomy (minutes)	37.15 ± 13.64	36.56 ± 13.10	0.922
Average cost of 3D Printing ($)	268.25	268.25	—

## 4 Discussion

Treating severe complex spinal deformities poses significant challenges, particularly in cases involving congenital malformations, multilevel fusions, and spinal rigidity, often necessitating Schwab grade 3 or higher osteotomies for adequate correction (Ramey et al., 2021). Osteotomy sites are typically near the apex of the deformity, adjacent to the spinal cord, increasing the risk of intraoperative neurologic injury. Lenke et al. reported that in a study of 147 patients with severe spinal deformities, the incidence of intraoperative neurophysiological events was 27.0%, and postoperative neurological complications occurred in 6.1% of cases (Lenke et al., 2013). Kelly et al. further noted a 9.9% rate of neurologic complications following three-column osteotomies (Kelly et al., 2014). The Scoli-RISK-1 study, an international multicenter prospective study involving 15 centers and 272 adult patients undergoing complex spinal deformity surgery, found that 23% of patients experienced reduced lower extremity motor scores at discharge (Lenke et al., 2018). In another study by Kim et al., postoperative neurological complications occurred in 17.1% of patients undergoing PVCR for severe spinal deformity, with 3.3% suffering permanent neurologic impairment (Kim et al., 2012). Similarly, Chen et al. (2020) reported that of 177 patients, 58 (32.8%) experienced intraoperative neurophysiological events, with 22 patients (12.4%) developing postoperative neurological complications. Therefore, precise and safe pedicle screw placement and osteotomy are paramount in reducing neurologic risks. With advancements in digital orthopedics, we now can precisely plan screw trajectories, angles, optimal screw diameters, and osteotomy designs and simulations. However, due to the anatomical complexity and proximity to the spinal cord and blood vessels in spinal deformities, translating virtual plans into clinical reality remains challenging.

In recent years, 3D printing has gained prominence in the medical field, enabling the construction of anatomical models based on reconstructed CT or MRI data and providing new methodologies for teaching and research (Salmi, 2021; Sheha et al., 2019; Zamborsky et al., 2019). Wu et al. (2015) evaluated the accuracy of 3D-printed spine models and demonstrated their ability to reproduce anatomical details accurately, representing complex spinal deformities without the health and safety concerns associated with cadaveric models. Increasingly, surgeons are employing 3D printing to facilitate personalized surgical planning (Ding et al., 2023; McLaughlin et al., 2022). Unlike CT navigation, which requires intraoperative scanning, and robotic-assisted systems, which necessitate setup time and potential intraoperative adjustments, 3D-printed guides can significantly reduce both surgical time and intraoperative radiation exposure without the need for additional imaging or adjustments. Additionally, 3D-printed guides are less technically demanding and integrate smoothly into surgical workflows, offering a relatively flat learning curve for surgeons accustomed to traditional methods.

In spinal deformity surgery, personalized 3D-printed pedicle screw guides have demonstrated superior accuracy compared to freehand techniques (Izatt et al., 2007; Luo et al., 2019; Pan et al., 2023). A meta-analysis by Lopez et al. (2021) reviewed 17 studies and found that pedicle screw accuracy was significantly higher in 3D-printed guide cohorts than in non-3D-printed cohorts (96.9% vs 81.5%, P < 0.001). The effectiveness of 3D-printed guides in osteotomy procedures has also been validated; Pijpker et al. (2018) reported that osteotomy templates provide guidance in the initial stages of pedicle subtraction osteotomy (PSO), particularly for resection of the posterior elements in planned asymmetric PSOs. Tu et al. (2019) successfully used CAD-assisted 3D-printed titanium templates in the correction of severe kyphosis. Xin et al. (2023) highlighted the accuracy of 3D-printed PSO guides in surgical simulations, confirming their utility in procedural planning. Although these studies indicate the feasibility of 3D-printed guides for screw placement and osteotomies in complex spinal deformities, to our knowledge, this is the first study comparing the accuracy, effectiveness, and cost of different designs of 3D-printed guides. This study not only provides new evidence for the application of 3D printing in orthopedic surgery but also serves as a valuable reference for future clinical practice and research.

In this study, pedicle screw placement accuracy did not differ significantly between the two guide groups. However, cortical breaches exhibited both similarities and differences, primarily occurring above the T9 level, which relates to the precision of guide-to-bone surface conformity. Upper thoracic segments have smaller vertebral bodies and pedicles, limiting the contact area for the guides. In the direct guide group, achieving precise screw insertion sometimes required sacrificing bone surface contact, resulting in fewer contact points and an increased likelihood of breaches. Additionally, smaller pedicles, a higher proportion of cortical bone, and entry points typically located on the sloped transverse process in thoracic pedicles (Fennell et al., 2014) may cause drill tip slippage on hard cortical surfaces in the indirect guide group, leading to entry point deviation and a higher risk of cortical breaches. To mitigate this, we recommend pre-drilling the entry site at high speed in the indirect group to secure anchoring while maintaining high RPM and slowly advancing the drill.

Furthermore, the direct guide group experienced two guide base fractures, likely due to stress from limited guide-bone contact during forceful application. It is important to note that this study used only skeletal spine models, which do not simulate muscles, ligaments, or other soft tissues. In actual surgeries, direct guides, due to their larger size, often require greater surgical exposure, especially in concave anatomical regions, which can hinder precise guide placement. Additionally, obstructive deformity ribs and residual soft tissue interposition between the guide and bone surface may further compromise screw placement accuracy.

Regarding osteotomy precision, there were no statistically significant differences between the direct and indirect groups in the coronal and sagittal planes (P = 0.433, P = 0.156), with both methods demonstrating similar accuracy. This may be due to the inability to simultaneously position the osteotomy guide and temporary rod, as current 3D-printed guides are primarily designed for the initial stage of three-column osteotomy, specifically posterior column resection, and positioning, thereby limiting guidance for middle and anterior column osteotomies. Therefore, the differences between the two groups were minimal, but both demonstrated a high level of design conformity.

In this study, the total design time for the direct group was significantly longer than for the indirect group (280.0 ± 73.03 min vs 206.2 ± 62.25 min). The design time per guide was also notably higher in the direct group (21.99 ± 3.16 min) compared to the indirect group (16.52 ± 2.12 min, P < 0.001). This difference is largely attributed to the thicker diameter of the direct group guides, which reduces the base surface area and increases the risk of errors in hollow sections of the model, necessitating more precise adjustments. Additionally, to preserve limited contact area and avoid interference with the spinous process, the direct group required re-linking the guide in CAD, ensuring it did not breach the guide walls, which further increased complexity and time.

The 3D printing duration was influenced by factors such as material, model height, and orientation strategy. In this study, all guides were fabricated using an SLA photopolymerization printer (UnionTech Lite 600) and photopolymer resin (Syn80) to create 1:1 scale models and surgical guides. The printing time for the direct group guides was 54.50 ± 6.39 min, significantly longer than the indirect group (37.69 ± 3.99 min, P < 0.001), primarily due to the larger size and greater height of the direct group guides. Among factors affecting 3D printing time, model height plays a decisive role. To reduce height, all guides were printed flat. However, with an internal diameter of 13 mm in the direct group *versus* 3 mm in the indirect group, along with height differences ranging from 7 to 16 mm to accommodate rotational deformities and guide connections, the direct group’s larger size contributed to its longer printing time.

In reviewing the literature, the production costs of 3D-printed surgical planning models vary widely. According to a recent review (Lopez et al., 2021), costs range from $175 to $5,400, depending on the material, design complexity, and model detail. Available materials include resin, polystyrene, polymers, PVC, polycarbonate, PLA, titanium, stainless steel, polyamide, and acrylic. Takemoto et al. (2016) reported that using titanium 3D-printed pedicle screw guides achieved a placement accuracy of 98.4%. Compared to polyamide plastic, titanium’s higher strength and durability reduce warping or bending during printing, thus enhancing guide precision. However, titanium costs five times more than polyamide plastic. Additionally, polymer-based 3D models can cost up to $2,500—substantially higher than alternative materials with similar functionality at lower costs. In this study, the average printing cost for both direct and indirect guides was $268.25 (based on the hospital’s uniform pricing). We suggest that material selection for 3D printing should consider local economic factors and resource availability. Given that guides are auxiliary tools, cost-intensive materials should not be prioritized when resins or other materials provide satisfactory placement accuracy, thereby avoiding unnecessary financial burdens on patients.

## 5 Limitations

The limitations of this study are as follows: (1) The currently available 3D printing materials are not yet capable of fully replicating the complex anatomical structure of the spine, especially failing to account for the impact of soft tissues such as nerves, muscles, and ligaments, and cannot dynamically simulate morphological changes after spinal orthopedics; (2) Although the execution of osteotomy has reached a high degree of precision, the existing guide plate system still has deficiencies in controlling the osteotomy range of the anterior and middle columns; (3) When dealing with extremely rotated vertebral bodies, placing screws in an almost horizontal direction faces the dual challenges of inadequate surgical field exposure and difficulty in fitting the guide plate.

## 6 Conclusion

Both direct and indirect 3D-printed guide plates can significantly simplify the operations of screw implantation and advanced osteotomy, improving the precision of screw placement and osteotomy; Direct guide plates are comparable to indirect guide plates in terms of accuracy, but they have clinical limitations such as extended design cycles, increased printing time, and enlarged surgical field exposure.

## Data Availability

The raw data supporting the conclusions of this article will be made available by the authors, without undue reservation.
